# A Collaborative Secure Localization Algorithm Based on Trust Model in Underwater Wireless Sensor Networks

**DOI:** 10.3390/s16020229

**Published:** 2016-02-16

**Authors:** Guangjie Han, Li Liu, Jinfang Jiang, Lei Shu, Joel J.P.C. Rodrigues

**Affiliations:** 1 Department of Information and Communication Systems, Hohai University, 200 North Jinling Road, Changzhou 213022, China; liulihhuc@gmail.com (L.L.); jiangjinfang1989@gmail.com (J.J.); 2 Changzhou Key Laboratory of Sensor Networks and Environmental Sensing, Changzhou 213022, China; 3 Guangdong Petrochemical Equipment Fault Diagnosis Key Laboratory, Guangdong University of Petrochemical Technology, Maoming 525000, China; lei.shu@ieee.org; 4 Instituto de Telecomunicações, University of Beira Interior, Portugal and University of Fortaleza (UNIFOR), Ceará 60811-905, Brazil; joeljr@ieee.org

**Keywords:** underwater wireless sensor networks, trust evaluation, collaborative secure localization

## Abstract

Localization is one of the hottest research topics in Underwater Wireless Sensor Networks (UWSNs), since many important applications of UWSNs, e.g., event sensing, target tracking and monitoring, require location information of sensor nodes. Nowadays, a large number of localization algorithms have been proposed for UWSNs. How to improve location accuracy are well studied. However, few of them take location reliability or security into consideration. In this paper, we propose a Collaborative Secure Localization algorithm based on Trust model (CSLT) for UWSNs to ensure location security. Based on the trust model, the secure localization process can be divided into the following five sub-processes: trust evaluation of anchor nodes, initial localization of unknown nodes, trust evaluation of reference nodes, selection of reference node, and secondary localization of unknown node. Simulation results demonstrate that the proposed CSLT algorithm performs better than the compared related works in terms of location security, average localization accuracy and localization ratio.

## 1. Introduction

Underwater Wireless Sensor Networks (UWSNs) have gained researchers’ much attention in the past few years due to their great potential utility in many applications, such as ocean resource exploration, marine environment monitoring, ocean target surveillance, submarine tracking and disaster prevention [1,2]. In these applications, sensor node’s accurate and reliable locations are required. In addition, some network system functions, e.g., network topology management and design of network communication protocols, also need sensor nodes’ location information to achieve. Thus, the secure localization problem becomes one of the most important and fundamental issues in UWSNs. Many localization algorithms have been proposed for UWSNs [3]. However, few of them take location reliability or security into consideration, while UWSNs are always deployed in unattended and even hostile environment. Ensuring node safety is a basic and essential knowledge to improve node location accuracy and reliability. Therefore, in this paper, we study both node security and localization accuracy in the proposed secure localization algorithm.

A large number of security mechanisms have been proposed to ensure safety of sensor nodes. Traditional mechanisms, e.g., cryptography and authentication, can well resist external attacks, but cannot eliminate insider attacks effectively. In addition, the traditional mechanisms are not energy efficient for energy and resource limited UWSNs. Relatively speaking, trust management has been recently suggested as an innovative and energy efficient security mechanism. Although trust management mechanisms are widely studied in Internet, Aodhoc networks, P2P networks, social networks and Terrestrial Wireless Sensor Networks (TWSNs) for network security [4,5], they cannot be directly applied to UWSNs due to the unique characteristics of underwater environment, e.g., inevitable node movement, variable propagation delay, instable acoustic channel, poor underwater link quality and limited channel bandwidth. These factors mainly impact the success communication rates and the accurate trust assessments of underwater sensor nodes. This highlights the fact that it is critical to design a new trust model for UWSNs considering the unique characteristics of the acoustic communication channel and underwater environment.

In this paper, we propose a Collaborative Secure Localization algorithm based on Trust model (CSLT) for UWSNs. CSLT first uses trust model to ensure node safety and avoid the influence from malicious nodes, which ultimately reduces unknown nodes’ localization error and enhances localization accuracy. Then, based on the collaboration of sensor nodes, localization ratio and localization accuracy can be further improved. The proposed CSLT consists of the following five sub-processes: trust evaluation of anchor nodes, initial localization of unknown nodes, trust evaluation of reference nodes, selection of reference node, and secondary localization of unknown node. In the first sub-processes, each anchor node pretends to be an unknown node to ask for localization and evaluate trust for each other. Only trusty anchor nodes can be used to localize unknown nodes. Then, the unknown nodes fail to be localized in the initial localization process can ask for secondary localization. Before the secondary localization, the trust values of reference nodes are calculated based on the cloud theory. Only trusty reference nodes are chosen to further localize the rest of unknown nodes until all the nodes are successfully localized.

The rest of this paper is organized as follows: Section 2 reviews existing related works. Section 3 presents the network model, the assumptions and the overview of CSLT. Section 4 describes the proposed trust model in detail. Section 5 gives a detailed analysis of simulation results. And finally, Section 6 makes a conclusion of this paper and discusses our future research works of secure localization in UWSNs.

## 2. Related Work

In our previous work [3], the current existing underwater localization algorithms are mainly classified into two categories: (1) stationary localization algorithms and (2) mobile localization algorithms. In this section, we first summarize several typical underwater localization algorithms, and then analyze existing research problems of them.

In stationary localization algorithms, all the sensor nodes are assumed to be static without influence from dynamic underwater environment. For example, in [6], an efficient Area Localization Scheme (ALS) is proposed for UWSNs, where anchor nodes send localization beacons with different energy levels to unknown nodes. The unknown nodes which receive beacons firstly record ID numbers of anchor nodes and the corresponding energy levels, then send the recorded information to sink node. After receiving the information from unknown nodes, the sink node tries to localize unknown nodes according to the corresponding energy level and the locations of anchor nodes. In [7,8], an Underwater Positioning Scheme (UPS) is proposed, which localizes unknown nodes by using 4 non-coplanar anchor nodes in three dimensional UWSNs. UPS does not require time synchronization because it uses TDoA (Time Difference of Arrival) technique to measure distances between sensor nodes. However, the localization delay of UPS is quite long, and the localization coverage is limited by the communication range of sensor nodes. In [9,10], an Underwater Sensor Positioning (USP) scheme is proposed in for sparse three-dimensional UWSNs. In USP, all the sensor nodes are equipped with pressure sensors, thus they can obtain their own depth positions. Then, based on the projection technique, the three dimensional localization problem can be transformed into its two dimensional counterpart. In order to solve the problem of low localization coverage in large-scare UWSNs, the researchers also put forward many solutions, for example, in [11,12], the strategy of unknown node upgrade is proposed in a Large-Scale Hierarchical Localization (LSHL) approach. That is, the successfully localized unknown nodes can be upgraded as anchor nodes to help neighbor unknown nodes with localization. This strategy can well solve the limited localization coverage problem; however, the main drawback of LSHL is that the location error is accumulated.

In real applications, an absolutely stationary network does not exist. The underwater sensor nodes always freely float with ocean current. Therefore, in many research works, ocean current and sensor mobility are taken into account in localization algorithms. For example, in [13], a Collaborative Localization Scheme (CLS) is proposed for mobile UWSNs, where unknown nodes collaborate with each other to determine their positions autonomously without using any anchor nodes. In addition, in mobile UWSNs, mobile anchor node, e.g., Dive and Rise (DNR) anchor nodes [14,15], Autonomous Underwater Vehicles (AUVs) [16,17], mobile detachable elevator transceiver (DET) [18], are adopted to improve localization performance. DNR anchor nodes and AUV equipments are equipped with GPS. They can first obtain their positions on the ocean surface, then sink into water and broadcast their positions to localize unknown nodes. In order to avoid position change from ocean flow, the anchor nodes periodically rise to the surface to update their position information. Mobile anchor nodes can move not only in the vertical direction, but also in the horizontal plane. For example, in [19], a Range-free scheme based on Mobile Beacons (RSMB) is proposed for UWSNs, where a mobile anchor node moves on the sea surface at a constant speed following the random way-point (RWP) model and broadcasts localization beacons for unknown nodes. The unknown nodes localize themselves based on the projection technique. In [20,21], a novel Underwater localization approach based on Directional Beacons (UDB) is proposed for UWSNs, where a mobile AUV moves according to a predefined route navigated by compass and sends directional beacons to localize unknown nodes. Compared with stationary localization algorithms, mobile ones are more suitable for dynamic UWSNs.

In above mentioned typical underwater localization algorithms, there are four problems: (1) Many localization algorithms require time synchronization. However, few of them research on the time synchronization problem; (2) Most existing algorithms ignore the influence of ocean mobility; (3) Some localization algorithms use mobile anchor nodes, but how to reasonably and effectively design the path planning of mobile anchor nodes is not resolved; (4) The problems of malicious attacks and the related security mechanisms are not considered in the localization process. In [22], Liu *et al*. firstly propose a Joint time Synchronization and Localization (JSL) algorithm. In JSL, the effect of depth information on the speed of sound and time synchronization is considered. However, in practical applications, the underwater acoustic velocity is not a constant, which varies with underwater environmental information, e.g., depth, temperature and salinity. In [23], Diamant *et al*. propose a new joint time synchronization and localization algorithm based on the uncertainty of acoustic velocity. However, the algorithm assumes that each sensor node is equipped with a directional navigation system, which is difficult to meet in practice. And in 2014, Diamant *et al*. further study the ranging and positioning problem in visual line of sight (LOS) and non line of sight (NLOS) environments [24]. Considering the mobility of underwater environment, Guo et al. firstly propose a novel Localization for Active-restricted UWSNs (LAR) [25]. In [26], Kim *et al*. propose a time Synchronization and Localization algorithm using Seawater Movement Pattern (SLSMP) to improve localization accuracy based on the unique characteristics of UWSNs, e.g., inevitable node movement and variable propagation delay. For the third problem, many path planning schemes of mobile anchor nodes have been proposed. For example, in [27], Han *et al*. firstly propose a path planning scheme based on triangle for a mobile anchor node to minimize the average localization error of unknown nodes. In [28], a cooperative path planning method is proposed for mobile UWSNs, which can be used to locate and navigate underwater mobile AUV; however this method is only suitable for tracking one AUV at a time. For the forth problem, the research of malicious attacks and security mechanisms in the process of localization is still at the initial stage. Few study has been worked on the secure localization problem and a lot of research problems need to be solved. That is why we study the secure localization problem in this paper. In the next section, we will present our proposed localization scheme CSLT in detail.

## 3. Collaborative Secure Localization Algorithm

In this section, we first introduce the network model, related assumptions and definitions, then describe collaborative secure localization mechanisms and present the CSLT algorithm, which includes the following five sub-processes: (1) trust evaluation of anchor nodes; (2) initial localization of unknown nodes; (3) trust evaluation of reference nodes; (4) selection of reference node; and (5) secondary localization of unknown node.

### 3.1. Network Model and Assumptions

**Figure 1:**
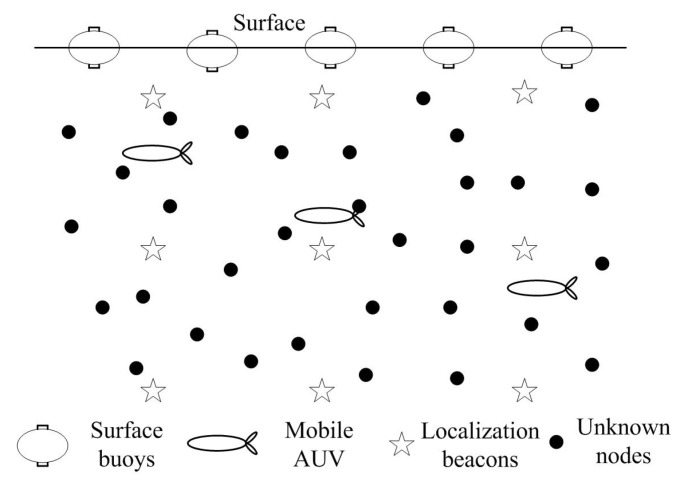
The structure of network model.

We assume there are three types of sensor nodes in the UWSNs: surface buoys, mobile AUVs, and unknown nodes, as shown in Figure 1. Surface buoys are floating on the ocean surface, which can directly obtain their positions by GPS and localize unknown nodes in their communication ranges. The AUV can first obtain its location on the surface, then dives into water to localization unknown nodes in the deep water. The sensor node which can first obtain its own location information and then help unknown nodes with localization is named as an anchor node. Therefore, both the surface buoys and the AUVs are anchor nodes. In this paper, we assume that all the anchor nodes can accurately obtain their positions from GPS as some previous underwater localization algorithms did. The GPS accuracy impacts on localization are not within the scope of this paper. All unknown nodes have the same initial energy level, the same capability of communication, computation and storage.

In the UWSN, there are *n* unknown nodes, denoted by $u_{i}\in S$, where $U={\left\{u_{i}\right\}}_{i=1}^{n}$. Each unknown node $u_{i}$ is randomly deployed at the position $p_{i}$ with the communication range *r*. Only when the distance $d(p_{i},p_{j})$ between two neighbor node $u_{i}$ and $u_{j}$ satisfies $d(p_{i},p_{j})\leq r$, the two neighbor unknown nodes can directly communicate with each other. We call them one-hop neighbor nodes. Similarly, there are two-hop and multi-hop neighbor nodes. In this paper, two-hop neighbor nodes will be used in the secondary localization. In addition, there are many malicious attacks in the process of localization, as summarized in Table 1. All the malicious attacks are compared and analyzed in terms of existing countermeasures, attack behaviors and attack results.

**Table 1:** Analysis on malicious attacks against localization in Underwater Wireless Sensor Networks (UWSNs).

Layers	Attacks	Countermeasures	Attack Behaviors	Results
Physical Layer	Stealing	The perception mechanism for physical damage, encryption algorithm, *etc*.	Signal eavesdropping and tampering	packet error and packet loss
	Jamming	Multi-frequency communication, using different transmission priority, *etc*.	Send jamming signal on the working frequency	packet loss
Data Link Layer	Collision	Forward Error Correct (FEC) code.	Repeat to send messages	packet loss
	Exhaustion	Limit the transmission speed and retransmission times of packets	Send a lot of useless messages	packet loss
	Unfairness	Avoid using long packets, redistributing transmission priority of packets, *etc*.	Deliberately take up the channel	packet loss
Network Layer	DoS attacks	Detection of energy consumption	Repeatedly send many messages to exhaust energy	packet loss
	Selective forwarding	Multi-path routing, reputation and trust model, *etc*.	Selectively forward packets	packet loss
	Sybil	Identity authentication of sensor nodes	Have multiple identities	packet error
	Wormhole	Construction of network topology	Shorten distance	packet error
	Sinkhole	Traffic monitoring, identity authentication, multi-path routing, *etc*.	Maliciously tamper with routing	packet loss
Transport Layer	Flooding	Limit the broadcast range of sensor nodes	Establish false connections	packet loss
	Tampering	Data encryption and node authentication.	Tampering localization beacons	packet error

### 3.2. Overview of Secure Localization Algorithm CSLT

Before introduce our proposed algorithm, some definitions are presented at first.

Definition 1. localization beacon. In the process of localization, anchor nodes broadcast information to help unknown nodes with localization. All the broadcasted packets, which include coordinate information, ID information, trust values, *etc*., are named as localization beacons.

Definition 2. upgrade anchor node. A successfully localized unknown node can work as an anchor node to localize neighbor nodes. In this paper, the trusty and successfully localized unknown node can be selected as an anchor node, we named it as an upgrade anchor node.

Definition 3. reference node. All the anchor nodes and upgrade anchor nodes are collectively named as reference nodes. They can help unknown nodes with localization.

In order to maximize localization ratio and improve localization accuracy of underwater sensor nodes, we propose a multi-anchor nodes collaborative localization (MANCL) algorithm in [29]. The MANCL algorithm divides the whole localization process into four sub-processes: (1) unknown node localization process; (2) iterative location estimation process; (3) 3D Euclidean distance estimation process; and (4) 3D DV-hop distance estimation process.

In the first sub-process, anchor nodes broadcast their coordinates periodically. All the unknown nodes that receive the coordinates can estimate their distances to the corresponding anchor nodes. If an unknown node receives four (or more than four) non-coplanar coordinates from different anchor nodes, the unknown node can calculate its position based on a multilateral localization method. However, in large-scale UWSNs, not all the unknown nodes can be successfully localized in the first sub-process. For example, some unknown nodes may not receive enough (at least 4) beacons from anchor nodes, or receive beacons from coplanar anchor nodes. In this case, the unknown nodes cannot be localized. In order to localize all the unknown nodes in the UWSN, we propose use other distance estimation methods and localization algorithms to help unknown nodes with localization.

First, the successfully localized unknown nodes are used to further localize their neighbor nodes in the second sub-process. The successfully localized unknown nodes with higher trust values can be chosen as upgrade anchor nodes; Then, the upgrade anchor nodes within two hops are used to help residual unknown node with localization during the rest of localization sub-processes; In the third sub-process, the improved 3D Euclidean distance estimation process which consists of two mechanisms (a communication mechanism and a vote mechanism) is proposed to localize unknown nodes. In the communication mechanism, non-localized unknown nodes use localized sensor nodes within communication range to estimate their coordinates. In the vote mechanism, neighboring anchor nodes and upgrade anchor nodes vote to determine the coordinates of non-localized sensor nodes. Finally, in the 3D DV-hop distance estimation process, the two-hop anchor nodes are used to calculate coordinates of unknown nodes. Simulation results show that, the proposed MANCL algorithm can perform better than related works with regard to localization ratio, average localization error, and energy consumption. However, how to evaluate trust values for sensor nodes are not discussed in [29]. Therefore, in order to improve location security of underwater sensor nodes, we propose a trust model for sensor nodes’ trust evaluation and apply the trust model in the secure localization.

**Figure 2:**
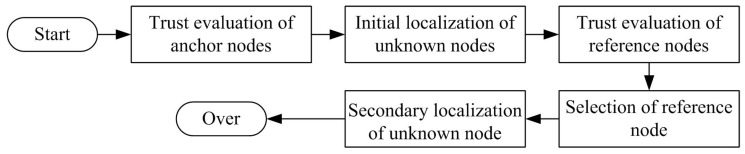
The five sub-processes in Collaborative Secure Localization algorithm based on Trust model (CSLT).

Based on our previous work MANCL in [29], the proposed secure localization algorithm in this paper consists of the following five parts: (1) trust evaluation of anchor nodes; (2) initial localization of unknown nodes; (3) trust evaluation of reference nodes; (4) selection of reference node; and (5) secondary localization of unknown node, as shown in Figure 2.

In the first sub-process, the trust values of anchor nodes are calculated based on the main idea of the reference [30] to detect malicious anchor beacons. The anchor node which sends legal localization beacons is assigned with a higher trust value. Only trusty anchor nodes can be used to localize unknown nodes in the rest of processes. In the second sub-process, the unknown nodes are localized based on the multilateral localization method by using positioning reference information from trusty anchor nodes. However, not all the unknown nodes can be successfully localized in the second sub-process. Therefore, in the third sub-process, we first evaluate trust value for each successfully localized unknown node. Then, the trusty and successfully localized unknown node can be selected as a reference node in the forth sub-process. Finally, in the fifth sub-process, we use two-hop trusty anchor nodes and reference nodes to help localize unknown nodes. How to calculate unknown nodes has been carefully discussed in [29]. Thus, in the next section, we mainly introduce how to evaluate trust values for sensor nodes, which is the most important part in the process of secure localization.

## 4. The Trust Evaluation Process

In this section, we introduce our proposed trust model in detail, including the overall architecture of the trust model, the process of trust calculation, trust combination and trust propagation. The structure of the trust model consists of four parts: (1) trust evidence generation; (2) trust calculation for one-hop neighbor nodes; (3) trust calculation for two-hop neighbor nodes; and (4) trust update.

**Figure 3:**
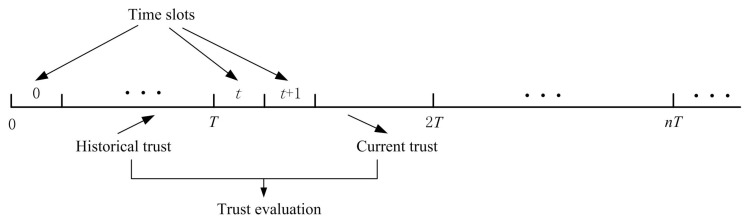
A sliding time window.

In UWSNs, sensor nodes always freely move with ocean current. It is generally known that the movement of current in seashore environments changes continuously and demonstrates certain semi-periodical properties. That is, the movement of sensor nodes in the ocean current also obeys certain semi-periodical properties. It is hard to describe the semi-periodical properties since they change with different underwater environment. For simplicity’s sake, we adopt a sliding time window concept [31] to periodically predict positions and calculate trust values for sensor nodes. Due to dynamic underwater environment, historical trust values should be taken into account to dynamically update trust relationship in time. In this paper, based on the sliding time window, we update sensor nodes’ trust values as follows. As shown in Figure 3, each time window *T* consists of several units *t*. During each time slot *t*, packets redare exchanged between two neighbor nodes, trust evidences can be collected to evaluate trust of sensor nodes. After a unit of time elapses, the window slides one time unit forward. Then, in the next time window t+1, the historical trust values can be used to update new trust values, thereby dropping the recorded information during the last time window *t*.

### 4.1. Trust Evidence Generation

In the first part of trust model, trust evidences are collected. As analyzed in the Table 1, there are many malicious attacks against localization and the main attack results can be classified into two categories: heavy packet loss and packet error. On one hand, malicious nodes can selectively forward or discard localization beacons, and they also can be selfish nodes refusing to take part in the localization process. In this case, unknown nodes may fail to localize themselves due to missing beacons, which introduces high packet loss. On the other hand, malicious nodes can monitor localization beacons from normal anchor nodes, then modify the beacons or send wrong localization information to mislead unknown nodes’ localization, which introduces high packet error. Therefore, packet loss and packet error are taken as two main trust evidences in the CSLT algorithm to detect malicious anchor beacons and calculate trust values of sensor nodes. If packet error of loss is detected, the related sensor nodes will be assigned with a lower trust values and will not be used in the localization process. The trust model is not used to detect malicious node, but used to choose sensor nodes with higher trust level to participate into localization to ensure localization ratio and accuracy. As a kind of intrusion tolerance technology, the trust model is used to ensure network security allowing existence of malicious node, which is a little different from the intrusion detection technology. The intrusion detection technology is always used to detect and remove malicious nodes. However, in the trust model, the malicious nodes are kept in the network while may not be used until they become trust ones.

At the beginning of localization, there are only three types of sensor nodes in the UWSN: surface buoys, mobile AUVs, and underwater unknown nodes. A real number from 0 to 1 is used to denote the trust value, 1 means completely trust and 0 means the opposite. We assume that all the anchor nodes are trusty at the beginning of network deployment, and set their trust value to 1. Then, based on the main idea of [30], we calculate trust value for anchor nodes according to packet loss and packet error. In the process of unknown nodes’ initial localization process, only one-hop anchor nodes are used. Therefore, we first introduce trust evaluation for one-hop neighbor nodes. Then, in the process of unknown nodes’ secondary localization process, two-hop anchor nodes and reference nodes are used. Thus, we will further present trust evaluation for two-hop neighbor nodes.

### 4.2. Trust Calculation for One-Hop Neighbor Nodes

In this subsection, the trust relationship between one-hop neighbor nodes is established by using the habit of human psychological cognition for reference, in which direct experience is the priority for evaluation and judgement. For example, in social network, if two people are friends or they are very familiar with each other, they can directly get assessment on each other’s trust level based on their daily communications. Otherwise, trust relationship between strangers needs recommendations from the third parties. Therefore, in the process of secure localization in UWSNs, if two neighbor anchor nodes have enough information for each other, that is there are enough trust evidences for evaluating trust relationship, we only need to calculate direct trust as final trust. Otherwise, only when the trust evidence is not sufficient for direct trust evaluation, neighbor nodes’ recommendations are required. This not only limits the number of information exchange between neighbor nodes, reduces the communication overhead and the complexity of the algorithm, but also greatly improves the accuracy of trust evaluation, and avoids the impact caused by malicious nodes in the process of trust recommendation, so that the trust evaluation between one-hop neighbor nodes is more true and reliable.

**Figure 4:**
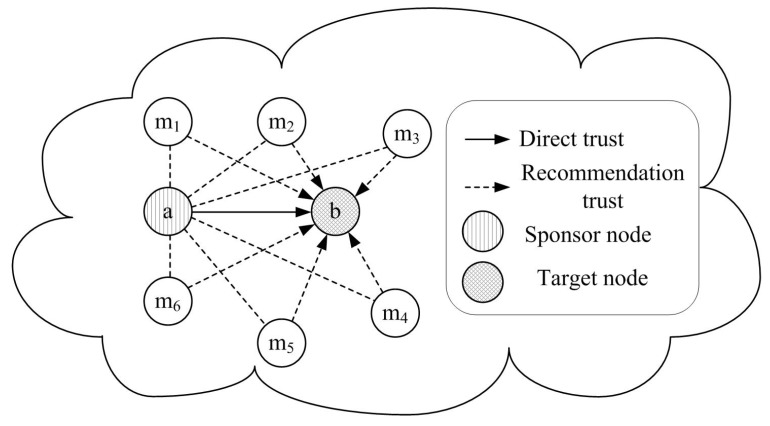
Trust calculation for one-hop neighbor nodes.

In the process of trust calculation for one-hop neighbor nodes, there are two main types of trust evaluation: direct trust evaluation and recommendation trust evaluation, as shown in Figure 4. If an anchor node **a** wants to obtain the trust value of another anchor node **b**, the evaluated anchor node **b** is named as a target node. The evaluating anchor node **a** which is responsible for trust evidence collection and trust evaluation is named as a sponsor node. Based on communication behaviors between anchor nodes, a sponsor anchor node can assign a target anchor node with different trust values. If there are direct communications between anchor nodes, direct trust is calculated. Otherwise, recommendation trust can be computed based on other anchor nodes’ recommendations. The anchor node which is selected as a trusted third party to provide recommendations is named as a recommender, just like $\left\{m_{1},m_{2},m_{3},\cdot \cdot \cdot \right\}$ in the Figure 4.

#### 4.2.1. Direct Trust Calculation for One-Hop Neighbor Nodes

If there are enough information interactions between neighbor nodes, direct trust can be calculated based on their communication behaviors. In this subsection, direct trust is calculated based on two trust evidences: packet error and packet loss. In current trust models, the packet loss is widely used as important trust evidence [32]. Thus in this paper, the statistics of packet loss is also used as follows.

It is generally known that underwater acoustic communications are characterized by high bit error and temporary loss of connectivity. Therefore, in UWSNs, the packet loss is always caused by three reasons: unreliable acoustic channel, inevitable node movement and malicious nodes. In order to accurately obtain the packet loss from malicious nodes, we first need to analyze the packet loss caused by acoustic communication channel and node movement. In the acoustic channel, the data packet loss is mainly caused by high Bit Error Rate (BER) which is directly related with the signal-to-noise ratio (SNR) of the channel. Therefore, the SNR of the acoustic channel is first analyzed as:

(1) $$SNR\left(l,f\right)=\frac{P/A(l,f)}{N(f)\Delta f}$$

where *P* is the acoustic signal power. Δf is the noise bandwidth of the receiver node. N(f) is the the overall power spectral density of noises. A(l,f) is the path loss of the acoustic signal. There are four kinds of the ocean background noise: turbulence, shipping, waves and thermal noise. We use $N_{t}\left(f\right)$, $N_{s}\left(f\right)$, $N_{w}\left(f\right)$ and $N_{th}\left(f\right)$ to denote the power spectral density of turbulence, shipping, waves and thermal noise, respectively.

(2) $$\left\{\begin{array}{c} 10\log N_{t}\left(f\right)=17-30\log f\\ 10\log N_{s}\left(f\right)=40+20\left(s-0.5\right)+26\log f\\ -60\log(f+0.03)\\ 10\log N_{w}\left(f\right)=50+7.5w^{1/2}+20\log f-40\log\left(f+0.4\right)\\ 10\log N_{t}h\left(f\right)=-15+20\log f \end{array}\right.$$

Therefore, we can obtain $N\left(f\right)=N_{t}\left(f\right)+N_{s}\left(f\right)+N_{w}\left(f\right)+N_{th}\left(f\right)$. In addition, the path loss A(l,f) is calculated by $A\left(l,f\right)=A_{0}l^{k}a{\left(f\right)}^{l}$, where $A_{0}$ is a normalization constant. *l* is the communication distance between two nodes. *k* is the energy propagation coefficient. In general, for the spherical spreading, k=2. a(f) is the absorption coefficient in underwater environment and *f* is the frequency of the acoustic signal.

Based on the SNR in Equation (1) and the modulation mode of acoustic communication, the average bit error rate (BER) can be calculated by $BER\left(l,f\right)=\Phi^{M}\left(SNR\left(l,f\right)\right)$, where *M* denotes the used modulation mode. Then, the packet loss caused by unreliable acoustic channel can be obtained as:

(3) $$P_{loss1}=\Psi^{F}\left(b,BER\left(l,f\right)\right)$$

where *F* denotes the forward error correction (FEC) mechanism. *b* is the length of the packet.

To study the packet loss caused by node movement, we adopt a Meandering Current Mobility (MCM) model [33] to describe the mobility of ocean current and underwater sensor nodes. MCM exploits the fact that the ocean current is a stratified, rotating fluid. Therefore, compared with the current movement in the horizontal direction, the vertical movement of ocean can be negligible. The ocean current is described as an incompressible two-dimensional flow, which is denoted by a stream function *ψ*. *ψ* consists of two components of the divergenceless velocity: $u=-\frac{\partial \psi }{\partial y},v=\frac{\partial \psi }{\partial x}$. *u* is the zonal (eastward) component of the velocity and *v* is the meridional (northward) one. The node movement is calculated based on the solution of Hamiltonian ordinary differential equations: $\dot{x}=-\partial_{y}\psi \left(x,y,t\right),\dot{y}=-\partial_{x}\psi \left(x,y,t\right)$. Therefore, we can judge whether two nodes **a** and **b** are neighbor nodes or move out of the communication range *r* according to the movement trajectory. If two node cannot directly communicate with each other, we think the packet loss is infinite. Other, if two sensor nodes are neighbors, the packet loss caused by node movement is 0. That is:

(4) $$P_{loss2}=\left\{\begin{array}{c} 0,d_{ab}<r\\ \infty ,d_{ab}\geq r \end{array}\right.$$

In each time slot, all anchor node broadcast information for their neighbor nodes and record the number of broadcasted packets *n*. The anchor nodes which receive the information also record the number of successfully received packets $n_{s-anchor}$. The number of all the loss packets is $n-n_{s-anchor}$. Because a part of packet loss is caused by unreliable acoustic channel $P_{loss1}$, the number of loss packets caused by malicious nodes should be $n_{f}=n-n_{s-anchor}-P_{loss1}$. Thus, the number of successfully received packets $n_{s}$ should be corrected as $n_{s}=n-n_{f}=n_{s-anchor}+P_{loss1}$. In the next time slot, if two neighbor nodes are still be neighboring, we obtain $P_{loss2}=0$. Otherwise, if two neighbor nodes move out of the communication range, we obtain $P_{loss2}=\infty$. In this case, the packet loss caused by malicious nodes $n_{f}$ is not updated and keeps the calculated value in the last time slot. Therefore, even if the receiver node cannot receive any packets, the sender node will not be erroneously assigned with a low trust value.

According to packet loss, we use the main idea of [34] based on Bayesian Formulation to evaluate trust relationship. In [34], the reputation of a sensor nodes is first calculated based on the beta distribution, which is indexed by two parameters (α,β) and can be expressed using the gamma function Γ(·) as $P\left(x\right)=\frac{\Gamma (\alpha +\beta )}{\Gamma (\alpha )\Gamma (\beta )}x^{\alpha -1}{\left(1-x\right)}^{\beta -1},\forall 0\leq x\leq 1,\alpha \geq 0,\beta \geq 0$. The reputation of node *j* evaluated by node *i* is given as $R_{ij}=Beta\left(\alpha_{j}+1,\beta_{j}+1\right)$, where $\alpha_{j}$ and $\beta_{j}$ represents the cooperative and non-cooperative interactions between node *i* and *j*, respectively. The trust of a sensor node is the statistical expectation of the reputation function and is calculated as:

(5) $$T_{ij}=E\left(R_{ij}\right)=E\left(Beta\left(\alpha_{j}+1,\beta_{j}+1\right)\right)=\frac{\alpha_{j}+1}{\alpha_{j}+\beta_{j}+2}$$

Therefore, based on *n* and $n_{s}$, and according to the reference [34], in which trust is denoted by $\left(\alpha_{j}+1\right)/\left(\alpha_{j}+\beta_{j}+2\right)$, the trust of an anchor node *j* can be calculated by node *i* as

(6) $$T_{ij}=\frac{n_{s}+1}{n_{s}+n_{f}+2}=\frac{n_{s}+1}{n+2}$$

In UWSNs, packet loss caused by unreliable acoustic channel and inevitable node movement is heavy. Getting statistics like packet loss as trust evidence is not reliable enough in underwater environment. Therefore, in this paper, we further propose using packet error to evaluate trust for sensor nodes based on the main idea of [30]. In [30], the main idea is to make use of the known locations of anchor nodes and the constraints that the locations must satisfy with the measurements (e.g., distance, angle) derived from the localization beacon signals. The anchor node which performs malicious node detection is named as detecting node. The detecting node first sends a localization request message to the target anchor node. The target node will send back a localization beacon packet which contains its own location information upon receiving the request message. Once the detecting node receives the beacon packet, it first estimates the distance between them. In addition, because the detecting node both knows its own location and receives the target node’s location, it can also calculate the distance between them. Finally, the detecting node compares the estimated distance and the calculated one. If the difference between them is larger than the maximum distance error, the detecting node can infer that the position information in the received beacon packet must be wrong. Similarly, in this paper, each anchor node broadcast their location information to neighbor nodes. They pretend to be unknown nodes to localize each other. According to the broadcasted coordinates, each anchor node can calculate the distances $l_{ij}$ to its neighbor nodes. At the same, the distances $l_{c}$ can be obtained by sound-ranging techniques, e.g., ToA (Time of Arrival), TDoA (Time difference of arrival), RToF (Round Trip time of Flight). For two neighbor anchor nodes *i* and *j*, we calculate their distance by

(7) $$l_{ij}=\sqrt{{\left(x_{i}-x_{j}\right)}^{2}+{\left(y_{i}-y_{j}\right)}^{2}}$$

If $|l_{ij}-l_{c}|>l_{th}$, where $l_{th}$ is the maximum ranging error, the received packet can be judged as including wrong coordinate information.

In UWSNs, the bit error rate (BER) of underwater acoustic channel is very high, thus the wrong coordinate information may be caused by BER. We cannot directly judge whether the neighbor node is trusty or not based on the result of a calculation from Equation (6). Therefore, we propose that node *i* record the number of correct packets and wrong packets from node *j* as $n_{c}$ and $n_{r}$, respectively. In each time period *T*, we obtain the number of neighbor node’s cooperative and non-cooperative behaviors as

(8) $$\left\{\begin{array}{c} n_{1}=n_{s}+n_{c}\\ n_{2}=n_{f}+n_{r} \end{array}\right.$$

Therefore, based on packet loss and packet error, the trust of node *j* is calculated by

(9) $$T_{ij}=\frac{n_{1}+1}{n_{1}+n_{2}+2}$$

In each time period *T*, node *i* can calculate trust value of node *j* for *N* time.

How to combine the *N* calculated trust values to evaluate the trust relationship is the essential problem to research in the trust model. In underwater environment, the acoustic communication and the behaviors of packet exchanges are uncertainty, thus the calculated *N* calculated trust values are different. The usually used average method and the median method cannot deal with the uncertainty characteristic of acoustic communication. Thus, in this subsection, we adopt cloud theory to calculate the sensor node’s direct trust based on the *N* trust values, since cloud theory are well used to describe uncertainties relationship, e.g., fuzziness and randomness [35]. For any trust attribute *x*, if ∀x∈X, where *X* is a trust evaluation domain denoted with accurate value, there is a mapping *μ* satisfies that

(10) μ:X→[0,1],x→μ(x)∈[0,1]

then, the distribution of *x* in the domain *X* is called trust cloud.

Cloud model has three digital features $\left(E_{x},E_{n},E_{e}\right)$. $E_{x}$ is the excepted value of the attribute, which reflects the central trust value. $E_{n}$ is the entropy of the attribute, which reflects the trust ambiguity of $E_{x}$. $E_{e}$ is the hyper entropy of the attribute, which reflects the uncertainty of $E_{n}$.

For any $x_{i}$, we can obtain $\left(E_{xi},E_{ni},E_{ei}\right)$ as follows:

- Step 1. Computing the mean value $\overset{-}{x_{i}}$ and the variance $S^{2}$ of $x_{i}$, $\overset{-}{x_{i}}=\frac{1}{n}\sum_{i=1}^{n}x_{i}$, $S^{2}=\frac{1}{n-1}\sum_{i=1}^{n}{\left(\overset{-}{x_{i}}-x_{i}\right)}^{2}$.
- Step 2. Computing $E_{xi}$, $E_{xi}=\overset{-}{x_{i}}$.
- Step 3. Computing $E_{ni}$, $E_{ni}=\sqrt{\frac{\pi }{2}}\times \frac{1}{n}\sum_{i=1}^{n}\left|\left.E_{xi}-x_{i}\right|\right.$.
- Step 4. Computing $E_{ei}$, $E_{ei}=\sqrt{S^{2}-{E_{ni}}^{2}}$.

Therefore, according to the *N* calculated trust values $T_{ij}$, we can obtain the direct trust $T_{d}\left(E_{x},E_{n},E_{e}\right)$ based on the excepted value, the entropy and the hyper entropy of the attribute $T_{ij}$.

#### 4.2.2. Recommendation Trust Calculation for One-Hop Neighbor Nodes

If there are not enough information interactions between two neighbor nodes, the trust relationship between them needs to be established based on the third party nodes’ recommendations. As shown in Figure 4, the first step of recommendation calculation is to select reliable recommender nodes among all the neighbor nodes. If node **a** wants to obtain the recommendations of node **b**, it first broadcasts recommendation request to all the common neighbor nodes of them. The neighbor nodes which have the trust value of node **b** will reply related acknowledgment packet to node **a**. The reliable recommenders are selected among the reply nodes according to the following principle: (1) the selected recommender should be trusty; (2) the recommendation value should be reliable. Whether a recommender is trusty or not can be easily judged by its trust value. The reliability of a recommendation value is decided by the familiarity degree between the recommender and the sponsor node. In general, people are more dependent on more familiar things and think they are reliable. Similarly, the more frequently two neighbor nodes communication with each other, the higher credibility of the recommendation trust value is. That is, the reliability of a recommendation value is judged by the familiarity among neighbor nodes.

If there are *m* recommenders selected for target node, we can obtain *m* recommendation values as $\left\{\left.T_{r1}\left(E_{x1},E_{n1},E_{e1}\right),T_{r2}\left(E_{x2},E_{n2},E_{e2}\right),\cdot \cdot \cdot ,T_{rm}\left(E_{xm},E_{nm},E_{em}\right)\right\}\right.$. Therefore, the total recommendation trust $T_{r}\left(E_{x},E_{n},E_{e}\right)$ can be calculated by

(11) $$T_{r}=T_{r1}⊕T_{r1}⊕\cdot \cdot \cdot ⊕T_{rm}$$

(12) $$T_{r}=T_{r1}\left(E_{x1},E_{n1},E_{e1}\right)⊕T_{r2}\left(E_{x2},E_{n2},E_{e2}\right)⊕\cdot \cdot \cdot ⊕T_{rm}\left(E_{xm},E_{nm},E_{em}\right)$$

(13) $$\left\{\begin{array}{c} E_{x}=\frac{1}{m}\prod_{i=1}^{m}E_{xi}\\ E_{n}=\min\left(\frac{1}{m}\sum_{i=1}^{m}E_{ni},1\right)\\ E_{e}=\min\left(\frac{1}{m}\sum_{i=1}^{m}E_{ei},1\right) \end{array}\right.$$

### 4.3. Trust Calculation for Two-Hop Neighbor Nodes

**Figure 5:**
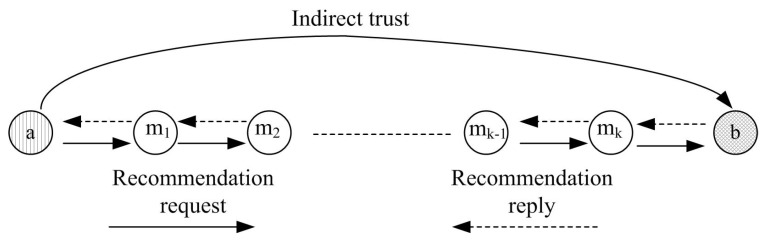
Trust calculation for two-hop neighbor nodes.

There are no direct interactions between non-neighbor nodes; therefore, the trust relationship between two-hop neighbor nodes needs to be established based on the trust recommendations from the third intermediate nodes. As shown in Figure 5, the trust evaluation process of non-hop neighbor nodes can be described as follows. First, for multi-hop neighbor node, several intermediates nodes are selected to obtain recommendation trust. The principle to select the multi-hop intermediates nodes is the same as the recommender selection in previous sub-section. Then, a trust routing path is established to propagate trust from the sponsor node to the target node. The trust attenuates along with the growing routing path. The longer the path is, the greater attenuation of trust is. Therefore, the shortest path should be selected to route recommendations. Finally, the indirect trust is calculated based on all the recommendations.

If there are *k* anchor nodes on the trust propagation path, we can obtain *k* recommendation values as $\left\{\left.T_{ind1}\left(E_{x1},E_{n1},E_{e1}\right),T_{ind2}\left(E_{x2},E_{n2},E_{e2}\right),\cdot \cdot \cdot ,T_{indk}\left(E_{xk},E_{nk},E_{ek}\right)\right\}\right.$. Therefore, the indirect trust $T_{i}nd\left(E_{x},E_{n},E_{e}\right)$ can be calculated by

(14) $$T_{ind}=T_{ind1}⊗T_{ind1}⊗\cdot \cdot \cdot ⊗T_{indk}$$

(15) $$T_{ind}=T_{ind1}\left(E_{x1},E_{n1},E_{e1}\right)⊗T_{ind2}\left(E_{x2},E_{n2},E_{e2}\right)⊗\cdot \cdot \cdot ⊗T_{indk}\left(E_{xk},E_{nk},E_{ek}\right)$$

(16) $$\left\{\begin{array}{c} E_{x}=\prod_{i=1}^{k}E_{xi}\\ E_{n}=\min\left(\sum_{i=1}^{k}{E_{ni}}^{2},1\right)\\ E_{e}=\min\left(\sum_{i=1}^{k}E_{ei},1\right) \end{array}\right.$$

In this paper, only two-hop anchor nodes and reference nodes are used in the secure localization, therefore, k=2. However, the proposed trust model is also suitable for other networks where k>2.

### 4.4. Trust Update

In order to timely and accurately update trust values, a new trust value $T_{new}$ is calculated based on the historical trust $T_{old}$ and the calculated trust in current time period $T_{now}$:

(17) $$T_{new}=\omega_{old}T_{old}+\omega_{now}T_{now}$$

where $\omega_{old}$ and $\omega_{now}$ are the weight values of $T_{old}$ and $T_{now}$, $\omega_{old}+\omega_{now}=1$. The two weight values are decided by the number of neighbor node’s cooperative behaviors. If the number of neighbor node’s cooperative behaviors in current time period $n_{1new}$ is higher than that in the last time period $n_{1old}$, we obtain $0.5\leq \omega_{old}<1,0\leq \omega_{1new}<0.5$. The historical trust is mainly used as reference for trust update in order to prevent malicious nodes from intentionally and rapidly increasing their own trust values by camouflage or lying. Otherwise, $0\leq \omega_{old}<0.5,0.5\leq \omega_{1new}<1$. The current calculated trust is mainly used as reference to punish the behaviors of malicious nodes.

## 5. Simulation Results and Discussions

In this section, the performance of the proposed secure localization algorithm is evaluated. The algorithm was implemented using MATLAB. In the experiments, the deployment area is set to 500 m × 500 m × 500 m. There are 500 unknown nodes randomly deployed in the 3D space. The communication range of unknown nodes is set to 100 m. Other parameters used in simulations are listed in Table 2. The performance of CSLT is compared based on the following three metrics: (1) detect ratio of malicious nodes, which is used to evaluate the security of CSTL; (2) localization accuracy, which is one of the most important evaluation indicators of localization algorithms; (3) localization ratio, which is used to evaluate whether all the unknown nodes can be successfully localized; (4) energy consumption, which is used to evaluate energy efficient of the proposed algorithm in energy-limited UWSNs.

**Table 2:** Simulation Parameters.

Parameters	Value
Simulation region size	500 m × 500 m × 500 m
The number of unknown nodes	500
Communication range	100 m
Node placement	Randomly deployed
Initial trust value	1
Acoustic channel bandwidth	100 Kbps
The modulation mode for acoustic communication	BPSK modulation
Mobility model	The Meandering Current Mobility (MCM) model

### 5.1. Comparison of Detect Ratio

**Figure 6:**
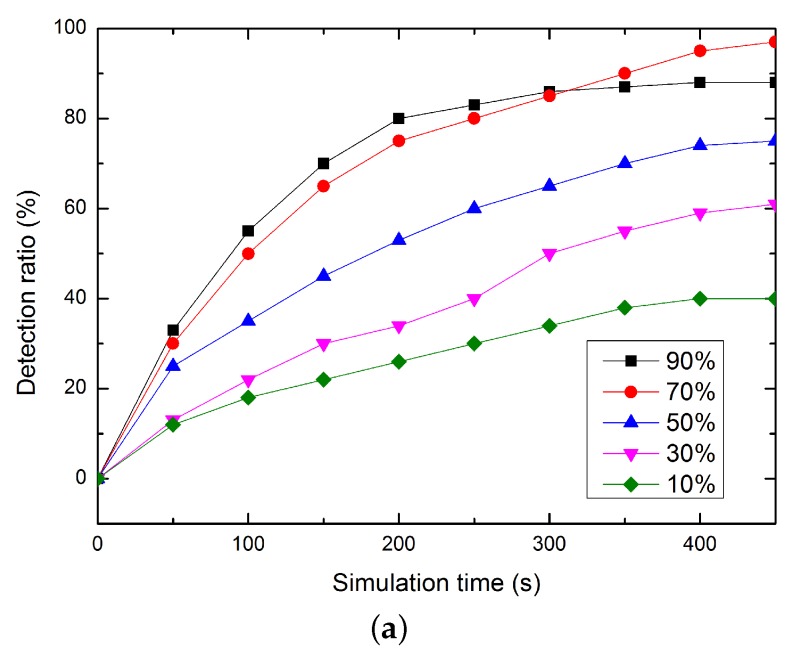

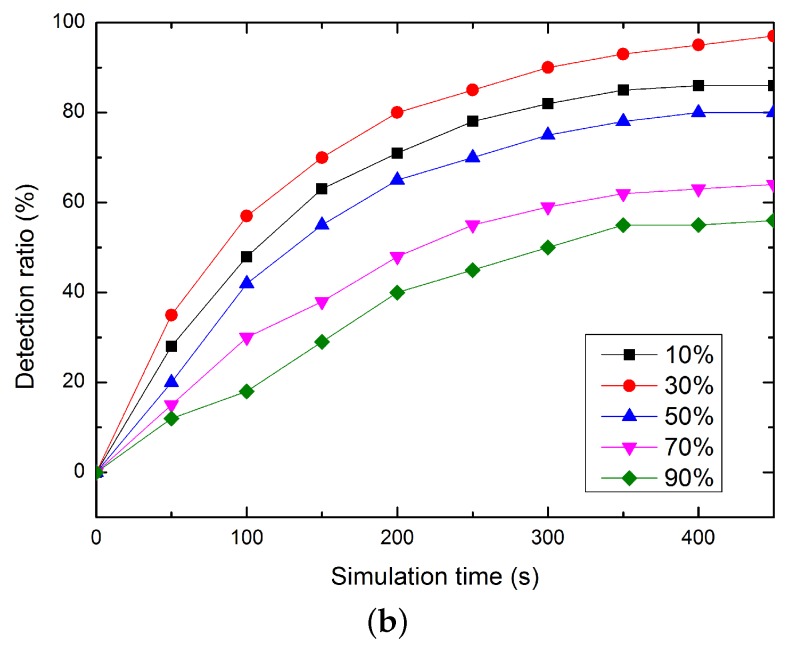
The performance of detection ratio. (**a**) $n_{1new}>n_{1old}$; (**b**) $n_{1new}<n_{1old}$.

In our proposed CSLT, the weight values chosen for trust update are not constants, which are decided based on the knowledgeable of current network situation and directly impact the performance of CLST. Thus, we first simulate the weight value’s on the algorithm performance, e.g., the detect ratio of malicious nodes. As shown in Figure 6, the detection ratio of malicious nodes rises step by step along the simulation progresses. When $n_{1new}>n_{1old}$, choosing $\omega_{old}$ as $0.5\leq \omega_{old}<1$ can detect more malicious nodes. This is because all the malicious nodes which intentionally to increase their values can be effectively detected. When $n_{1new}<n_{1old}$, choosing $\omega_{old}$ as $0\leq \omega_{old}<0.5$ can performance better. This is because, in this case, mainly using the current calculated trust as reference to update trust can well punish the behaviors of malicious nodes.

**Figure 7:**
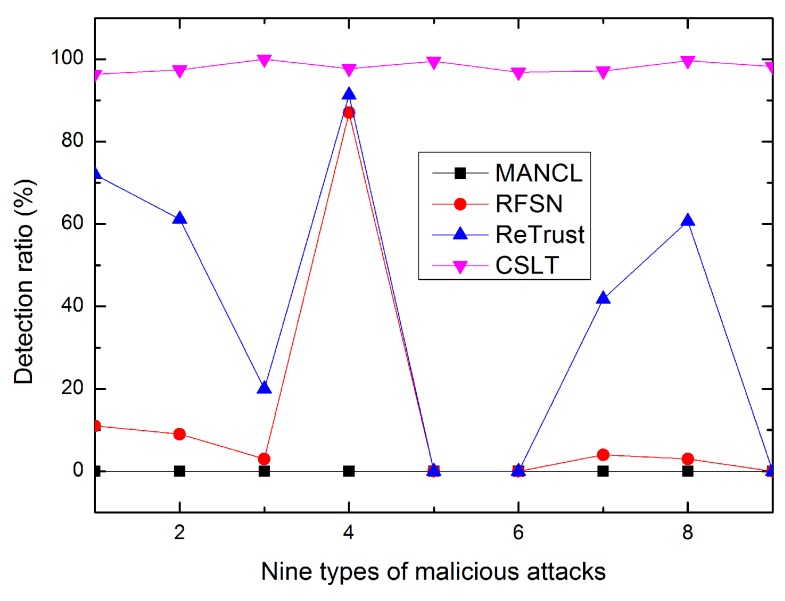
Comparison of detect ratio.

Our secure localization is proposed based on MANCL [29], ReTrust [31], and RFSN [34], thus CSLT is compared with the three references. As shown in Figure 7, nine types of malicious nodes are detected. They are (1) signal interference attacks; (2) link collision attacks; (3) DoS attacks; (4) selective forwarding attacks; (5) Sybil attacks; (6) wormhole attacks; (7) sinkhole attacks; (8) flooding attacks and (9) packet tampering attacks. Simulation results show that our proposed CSLT can well detect the nine types of malicious nodes with more than 95% detection ratio. However, we cannot find malicious node with 100%. This is because some malicious nodes which cause packet error or loss of the surrounding nodes’ instead of in their own communication cannot be efficiently found out. MANCL cannot detection all the malicious attacks because it does not use any secure mechanism in the process of localization. RFSN and ReTrust are only effective against selective forwarding attacks, since they just consider packet loss to evaluate trust for sensor nodes. Therefore, they cannot detect the rest types of malicious nodes, e.g., Sybil attacks, wormhole attacks and packet tampering attacks. However, ReTrust works better than RFSN in terms of malicious node’s detection ratio. This is because in RFSN, only direct trust are calculated and only one-hop neighbor nodes’ behaviors can be monitored, while in ReTrust, neighbor nodes’ recommendation are also taken into account to evaluate trust. Relatively specking, ReTrust is much more reliable and robust against malicious attacks than RFSN.

### 5.2. Comparison of Localization Accuracy

**Figure 8:**
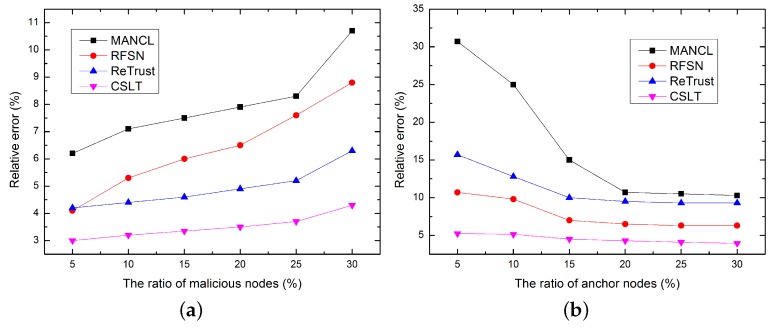
Comparison of localization accuracy. (**a**) Impact of malicious node ratio; (**b**) Impact of anchor node ratio.

Localization accuracy is one of the most important evaluation indicators of localization algorithms. In this paper, the localization accuracy is denoted by the relative error between the actual location and the calculated node position. As shown in Figure 8a, the ratio of malicious nodes varies from 5% to 30% with increments of 5%. The impact of malicious node ratio on localization accuracy is first evaluated. Simulation results show that localization errors increases with the increasing number of malicious nodes, since more malicious nodes introduce more attacks and interferences on localization. However, CSLT still out performs other algorithm in terms of localization accuracy.

In addition, the impact of anchor node ratio on localization accuracy is evaluated in Figure 8b. Simulation results show that with the increase number of anchor nodes, the average localization errors of all the algorithms decrease rapidly. This is because more localization beacons can be provided by more anchor nodes to localize unknown nodes. Most unknown nodes can be successfully localized in the initial localization process. Only a small part unknown nodes need to be localized in the secondary localization process. The accumulated localization errors from upgrade anchor node can be greatly reduced, which ultimately improves localization accuracy.

### 5.3. Comparison of Localization Ratio

**Figure 9:**
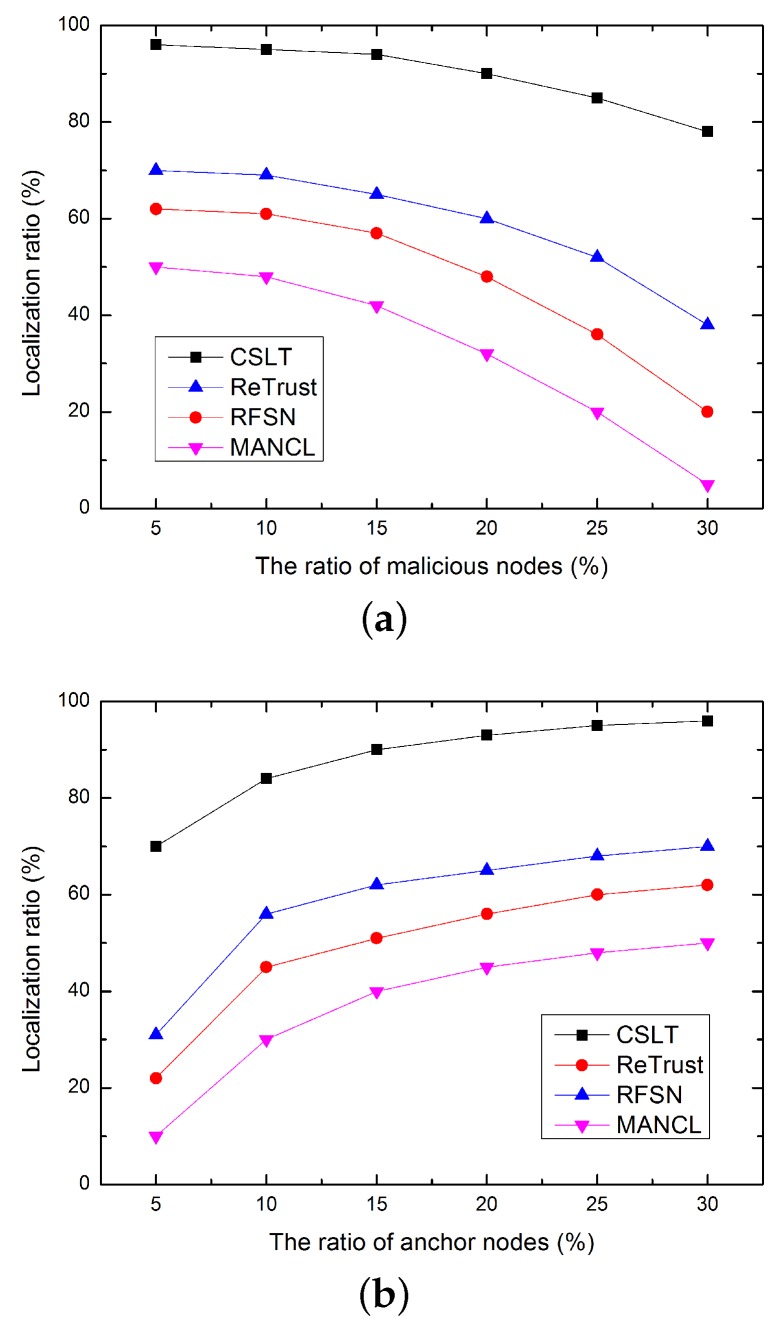
Comparison of localization ratio. (**a**) Impact of malicious node ratio; (**b**) Impact of anchor node ratio.

In this sub-section, the localization ratio is compared, which is defined as the percentage of successfully localized unknown nodes. As shown in Figure 9, the localization ratio decreases as the increasing number of malicious nodes, and increases with the growing number of anchor nodes. Obviously, more unknown nodes can be localized with more anchor nodes. In addition, more packet error and packet loss will be introduced by more malicious nodes. In this case, some unknown nodes cannot receive enough localization beacons to localize themselves, which greatly reduces the localization ratio. However, our proposed CSLT has higher localization ratio than the compared algorithm, this is because, in CSLT, a reliable trust model is proposed to ensure the security of sensor nodes, and a secondary localization process is also helpful to improve localization ratio.

### 5.4. Comparison of Energy Consumption

**Figure 10:**
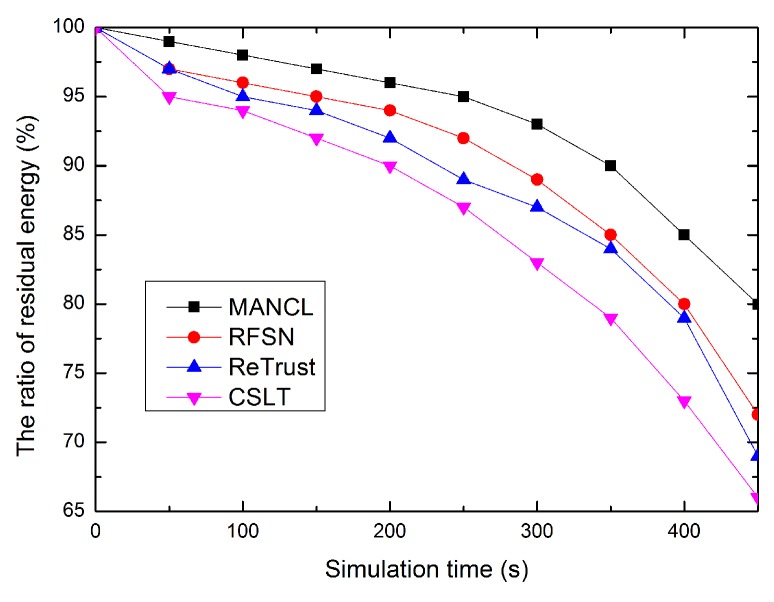
Comparison of energy consumption.

Finally, the energy consumption of each algorithm is compared. As shown in Figure 10, our proposed CSLT consumes more energy than the compared algorithms, e.g., MANCL, ReTrust, and RFSN. In order to detect malicious nodes, CSLT needs to collect trust evidences and calculates trust values for sensor nodes, which introduces more communication overheads and consumes more energy. However, CSLT can well protect the network and ensure node security. In addition, CSLT performs better than the three compared related works in terms of location security, average localization accuracy and localization ratio. That is to say, we sacrifice some energy to improve network security, and ultimately to improve performance of secure localization.

## 6. Conclusions

In this paper, we propose a collaborative secure localization algorithm based on the trust model for UWSNs. CSLT includes five sub-processes: trust evaluation of anchor nodes, initial localization of unknown nodes, trust evaluation of reference nodes, selection of reference node, and secondary localization of unknown node. Based on the trust model, the trust values of the one-hop anchor nodes and the two-hop reference nodes are calculated. Then, only trusty anchor nodes and references nodes are selected to localize unknown nodes to avoid impact from malicious nodes. Simulation results indicate that CSLT can achieve a high detect ratio of malicious nodes. In addition, the localization security including localization accuracy and localization ratio is improved in UWSNs. However, there are many remaining issues that need to be further studied for secure localization in UWSNs. First, we cannot find each type of malicious nodes with 100%. For example, some malicious nodes may not mostly cause packet error or loss in their own communication but the surrounding nodes’. Is this case, we just use the trust model to avoid the attacks from the malicious nodes. The influenced surrounding nodes are assigned with lower trust values, since their communication behaviors are heavily impacted by surrounding real malicious node. They are not chosen to localize to unknown node to avoid the influence from nearby malicious node. However, the real malicious nodes cannot be detected efficiently. In our simulation, the number of this kind of malicious node is relatively small. If most malicious nodes launch this kind of malicious attack, our trust model may not work very well. So we will further study new trust model to solve this problem in the future research. In addition, we use MATLAB to evaluate the performances of the proposed algorithm in order to easily compare our new algorithm with our previous work in [29]. To make our simulation results more reliable, we will further study the new platforms, e.g., NS, Aquasim, etc., for underwater environment. Last but not least, we assume that all the anchor nodes can accurately obtain their positions from GPS as some previous underwater localization algorithms did. While in real application, it is hard for the anchor nodes to accurately obtain their positions from GPS, and the position accuracy of anchor nodes is heavily influence localization accuracy of unknown nodes. Therefore, we will further to study the GPS accuracy impacts in our future work.
